# The Princeton Protein Orthology Database (P-POD): A Comparative Genomics Analysis Tool for Biologists

**DOI:** 10.1371/journal.pone.0000766

**Published:** 2007-08-22

**Authors:** Sven Heinicke, Michael S. Livstone, Charles Lu, Rose Oughtred, Fan Kang, Samuel V. Angiuoli, Owen White, David Botstein, Kara Dolinski

**Affiliations:** 1 Lewis-Sigler Institute for Integrative Genomics, Princeton University, Princeton, New Jersey, United States of America; 2 The Institute for Genomic Research, Rockville, Maryland, United States of America; 3 Center for Bioinformatics and Computational Biology, University of Maryland, College Park, Maryland, United States of America; Utrecht University, Netherlands

## Abstract

Many biological databases that provide comparative genomics information and tools are now available on the internet. While certainly quite useful, to our knowledge none of the existing databases combine results from multiple comparative genomics methods with manually curated information from the literature. Here we describe the Princeton Protein Orthology Database (P-POD, http://ortholog.princeton.edu), a user-friendly database system that allows users to find and visualize the phylogenetic relationships among predicted orthologs (based on the OrthoMCL method) to a query gene from any of eight eukaryotic organisms, and to see the orthologs in a wider evolutionary context (based on the Jaccard clustering method). In addition to the phylogenetic information, the database contains experimental results manually collected from the literature that can be compared to the computational analyses, as well as links to relevant human disease and gene information via the OMIM, model organism, and sequence databases. Our aim is for the P-POD resource to be extremely useful to typical experimental biologists wanting to learn more about the evolutionary context of their favorite genes. P-POD is based on the commonly used Generic Model Organism Database (GMOD) schema and can be downloaded in its entirety for installation on one's own system. Thus, bioinformaticians and software developers may also find P-POD useful because they can use the P-POD database infrastructure when developing their own comparative genomics resources and database tools.

## Introduction

With the great explosion of biological data in the last decade, biological databases have become an essential part of today's research. The earliest online databases were the sequence repositories, such as Genbank [1] and EMBL [2], that provided the non-expert public access to the sequence data for genes, chromosomes, and eventually entire genomes, along with highly effective query and comparison tools. Soon after, several model organism databases that store and display the annotated genome sequences of well-studied organisms were developed. These databases now serve as an essential basic information source for all kinds of biological researchers.

For working biologists, some of the most important information concerns the phylogenetic relationships among proteins, which is not necessarily straightforward to recover from the basic sequence databases. Regardless of which organism one works with, much of the functional annotation of gene and protein functions is transferred, based on sequence similarity, from other organisms where more experimental information is available (for example, see the Gene Ontology annotations at http://www.geneontology.org/GO.current.annotations.shtml). It is for this reason that sequence similarity searching has become one of the most popular database tools in current use, perhaps second only to searching the published literature. To make good use of sequence similarity information, it would be very useful to have a simple, user-friendly way to visualize relationships in their phylogenetic context, particularly the relationships among the proteins in the model organisms from which most of the functional annotations are derived. It is of particular value to be able to know which proteins are (or might be) orthologous [i.e. similar to each other in sequence because they originated from a common ancestor, having been separated in evolutionary time only by speciation event(s)]. It is also useful to see these orthologous relationships in the context of the larger paralogous gene families ultimately caused by gene duplications during the course of evolution.

In this paper, we describe P-POD, which provides the user an easy way to find and visualize the orthologs to a query sequence in the eukaryotes of greatest interest to working biologists (*i.e.* the experimental model organisms and the human) in their evolutionary context, and to link these relationships with the relevant literature. Several databases that specialize in comparative genomics have recently come online. Each of these databases, including P-POD, has both useful features and problems specific to the methods or species chosen in the analysis (Table 1, reviewed in [3]); none is perfect, but each fulfills the needs of particular database users.

**Table 1:** Comparative genomics web resources.

Name	Description	Ortholog prediction	Larger seq. families	Disease information	Curated literature
Clusters of Orthologous Groups (COGs/KOGs) [22]	Provides groups of orthologous proteins for seven eukaryotic species; the construction protocol involves manual curation	Yes	Yes	No	No
Eukaryotic Gene Orthologs (EGO) [23]	Displays predicted orthologs derived from several eukaryotic genomes based on gene alignments	Yes	No	No	No
Homologene [24]	Provides automated predictions of homologs among the genes of several eukaryotes	No	Yes	Yes	No
Inparanoid [25]	Houses pair-wise groups of orthologous proteins for multiple species	Yes	No	No	No
OrthoDisease [26]	Uses the Inparanoid algorithm to generate pair-wise orthologs between human disease genes and genes from other species	Yes	No	Yes	No
OrthoMCL-DB [4], [27]	Utilizes a Markov Cluster algorithm to predict orthologous groups of proteins for multiple species simultaneously	Yes	No	No	No
Sybil (S. Angiuoli and O. White, in preparation)	Uses Jaccard clustering to group sequences based on pair-wise BLAST analysis	No	Yes	No	No
YOGY [28]	Retrieves orthologous proteins from four different resources: KOGs, Inparanoid, Homologene, and OrthoMCL-DB	Yes	No	No	Yes (only budding and fission yeast)
P-POD (This study)	Orthologs and Jaccard clusters	Yes	Yes	Yes	Yes

P-POD is meant to complement these existing databases by providing a comparative genomics analysis system readily accessible to and readable by experimentalists, containing not just computational comparative analyses of the most common experimental organisms but also literature curation and links to other databases of interest. For example, while the OrthoMCL database contains sequences from over 55 prokaryotic and eukaryotic genomes, we chose to include protein sequences from eight eukaryotic organisms for their medical value or their status as widely-studied model organisms. There are certainly users who would need the more comprehensive species set from OrthoMCL. While P-POD uses the underlying OrthoMCL algorithm, it is meant to complement the OrthoMCL online database by serving another set of users, primarily experimental biologists who wish to query with their gene of interest from a well studied model organism to quickly get the evolutionary context of that gene along with other relevant information about that gene without sorting through a very large list of other sequences.

We designed our comparative genomics analysis system so that different components could be added to and removed from the pipeline in a modular fashion; the initial version of the pipeline described here generates related protein families using two different methods to provide complementary views of phylogenetic relationships. We used OrthoMCL ([4]) to find the orthologs and a version of Jaccard Clustering [modified to find homologs across multiple genomes (S. Angiuoli and O. White, in preparation)] to provide a larger protein family context. The phylogenetic relationships among family members from each method are determined using CLUSTAL W [5] and PHYLIP and visualized as arbitrarily rooted trees. In addition, we provide relevant gene and disease information from the Online Mendelian Inheritance in Man (OMIM) [6] database and also provide information culled from the literature that can be used to indicate when functional conservation has been shown experimentally between predicted orthologs. All the data within the database are freely available through the web and by downloading the entire software and database system via the following URL: http://ortholog.princeton.edu/

Historically, genomic databases have been developed in isolation, with idiosyncratic database schemata and software. Much duplication of effort can be avoided by developing generic modular databases and software that save, especially in the long run, both time and money spent on development, maintenance, and user training. In constructing P-POD we made use of the database schema, installation and loading tools, and various software components from the Generic Model Organism Database (GMOD) project (www.gmod.org), The goal of GMOD is to develop an open and generic genomic database environment, including database schemata and required software tools.

## Results

### The P-POD Pipeline

**Figure 1:**
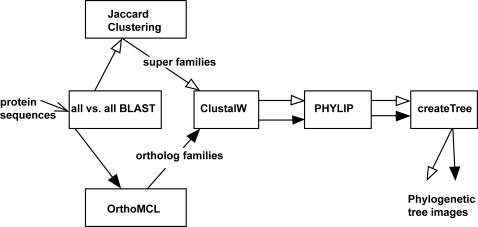
Steps in the analysis pipeline.

In the interests of both simplicity and flexibility, the P-POD pipeline employs a modular architecture. The pipeline takes FASTA-formatted protein sequences as input, performs comparative genomic analyses, and stores the results in a database. In addition, we have created web tools that allow searching and browsing of the results in a user-friendly manner. We built the initial pipeline to identify putative orthologous proteins using OrthoMCL [4]. We chose OrthoMCL over other algorithms mainly because it can be run on multiple species at once and is one of the better-performing algorithms in terms of sensitivity and specificity [7] [3]. We generated larger families of related sequences using Jaccard clustering modified to find homologs across multiple genomes; see the Materials and Methods section for algorithm details. It is important to note that we built the P-POD system so that we can easily add or remove results from different analysis methods. We acknowledge that the first choice is not always the best choice, and as algorithms improve and/or as users request other methods, we plan to modify and expand the system as appropriate. P-POD generates phylogenetic trees from both analyses using CLUSTAL W [5] and PHYLIP; the trees are graphically displayed on the web. The overall pipeline is illustrated in Figure 1. The sources and versions of the pipeline components are listed in Table 2. The data are stored in a Generic Model Organism Database (GMOD) database schema using the freely available PostgreSQL software to make the entire system accessible to as many users as possible, not only through the web but also via download of the entire system.

**Table 2:** Components of the analysis pipeline.

Program	Version	Source
GMOD::Loader		This study
WU-BLAST	2.0MP-WashU 10-May-2005	http://blast.wustl.edu/
OrthoMCL [4]	Version 1.2 14-March-2005	http://sourceforge.net/projects/orthomcl/
MCL [29]	Version 1.005, 05-118	http://micans.org/mcl/
Jaccard Clustering	NA	S. Angiuoli and O. White (in preparation)
Clustal W [5]	Version 1.83	ftp://ftp.ebi.ac.uk/pub/software/unix/clustalw
PHYLIP	Version 3.64	http://evolution.genetics.washington.edu/phylip.html
createTree		This study

**Figure 2:**
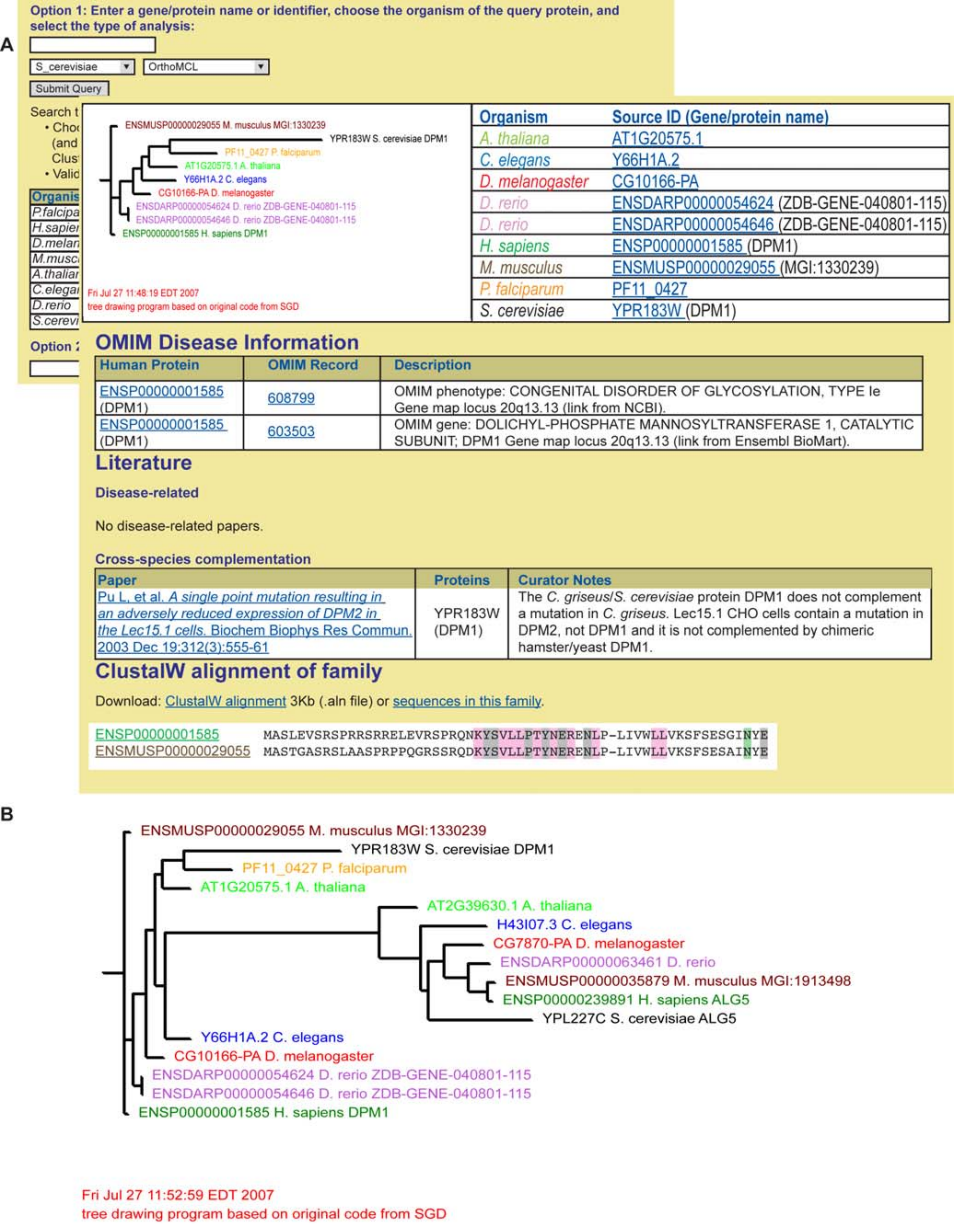
Screenshots of the P-POD web interface. (A) A portion of the results page for the *DPM1* OrthoMCL family is shown superimposed on the search form. Results from OrthoMCL are provided, and a link to the larger Jaccard family (B) is also available. Disease information from OMIM is displayed, as well as any relevant disease or cross-complementation literature.

The P-POD database contains protein sequences from eight eukaryotic organisms with fully sequenced genomes chosen for either their medical value or their status as widely-studied model organisms. They include a yeast (*Saccharomyces cerevisiae*), a nematode worm (*Caenorhabditis elegans*), a fruit fly (*Drosophila melanogaster*), a flowering plant (*Arabidopsis thaliana*), a fish (*Danio rerio*), a mouse (*Mus musculus*), and human (*Homo sapiens*). These are the leading experimental organisms for modern biologists, and among them span much of the evolutionary tree of the eukaryotes. Also included is the malaria parasite *Plasmodium falciparum*, an organism that, although it is a eukaryote, has a relatively exotic parasitic lifestyle. Sources for each protein set are listed in Table 3. Also stored in the system are results from each step of the pipeline, gene and disease information from OMIM, and curated information from the literature describing experimental tests of functional conservation (see Figure 2).

**Table 3:** Sources and numbers of sequences analyzed.

Organism	Proteins	Database	Filename
*S. cerevisiae*	6704	SGD	orf_trans_all.fasta.gz
*H. sapiens*	33869	ENSEMBL	Homo_sapiens.NCBI35.nov.pep.fa.gz
*M. musculus*	36471	ENSEMBL	Mus_musculus.NCBIM34.nov.pep.fa
*D. rerio*	32143	ENSEMBL	Danio_rerio.ZFISH5.nov.pep.fa
*D. melanogaster*	19178	FlyBase	dmel-all-translation-r4.2.1.fa
*C. elegans*	22858	WormBase	wormpep150.fa
*A. thaliana*	30690	TAIR	TAIR6_pep_20051108.fa
*P. falciparum*	5363	PlasmoDB	Pfa3D7_WholeGenome_Annotated_PEP_2005.2.11.fa

The pipeline generated a total of 25,271 OrthoMCL families and 15,050 Jaccard Clustering families that contain a total of 165,970 proteins (154,736 and 152,799 for each method, respectively) from eight different organisms. There are 984 OrthoMCL families that contain at least one protein from each of the species, with 112 of them containing exactly one protein from each. We used the GO Term Mapper tool available at SGD to determine the distribution of GO annotations for the 112 yeast proteins in these families; we chose the yeast proteins because complete GO annotation is available for the entire yeast genome [8]. Not surprisingly, these proteins are involved in core biological processes that are common across eukaryotes, including translation, transport, cell cycle regulation, and cytoskeleton organization. These genes are also well characterized; only four of the 112 genes were annotated to “biological process unknown.” We also used the GO Term Finder [9] implementation at Princeton (http://go.princeton.edu/) to look for enrichment of GO terms among the 112 genes. Again unsurprisingly, the most significant shared term is “ribosome biogenesis and assembly” (corrected P-value = 5.85e-18) along with other terms related to translation and basic metabolic processes, all processes common among the eukaryotes.

The complete species distribution of each family is available via the web (http://ortholog.princeton.edu/organismdist.html), and the number of proteins found in families and orphan proteins (those not found in an OrthoMCL or Jaccard family) from all the species is found in Table 4.

**Table 4:** Number of proteins in each organism found in OrthoMCL or Jaccard families.

Organism	OrthoMCL	Jaccard	Orphan (% of total proteome)
*S. cerevisiae*	4,333	3,660	2,176 (32%)
*H. sapiens*	27,606	29,315	3,193 (9%)
*M. musculus*	29,214	31,388	3,902 (11%)
*D. rerio*	27,602	28,968	1,903 (6%)
*D. melanogaster*	16,015	15,048	2,503 (13%)
*C. elegans*	18,070	16,308	4,078 (7%)
*A. thaliana*	27,987	25,819	2,279 (13%)
*P. falciparum*	3,909	2,293	1,284 (33%)

The percentage of orphans is generally strikingly low, with the percent orphaned in a given species 13% or lower, with two exceptions, yeast (32%) and *Plasmodium* (33%). These numbers confirm the high conservation of proteins across eukaryotes, with the notable exception the *Plasmodium* outlier. The high percentage of yeast orphans is due to the fact that we did the analysis with the complete protein set, including over 800 ORFs flagged as “Dubious” by SGD; these are not likely to actually encode proteins, and when they are excluded the percentage of orphans in yeast drops to about 20%.

P-POD includes 1,895 human proteins that are associated with human diseases (based on protein-OMIM disease files downloaded from ENSEMBL), 1,852 of which were found in either an OrthoMCL or Jaccard family; in each of these cases, links to the relevant OMIM records are provided online.

### Manually Curated Information

P-POD also includes curated literature that contains information relevant to the yeast proteins in the database. The source of the literature is the *Saccharomyces* Genome Database (SGD). SGD provides a Literature Guide tool that categorizes yeast literature into different topics, two of which, “Cross-species expression” and “Disease-gene related,” are particularly relevant to the data in P-POD; we believe that this set of papers, which is continually updated and curated, contains most, if not quite all, of the experimental data testing functional conservation between yeast and other organisms. All papers associated with these topics were downloaded from the SGD FTP site and loaded into the database (see Materials and Methods). They are then displayed on the web interface, with links to PubMed, so that users can compare experimentally determined functional conservation and computationally predicted orthology. This set of papers does not, of course, address proteins without a yeast ortholog. A way of dealing with this limitation is under study; a likely development will be the inclusion of papers from the literatures of other model organisms. For disease-related genes, we provide OMIM links that at least partially fill this gap for the human.

In addition, we manually curated the “Cross-species expression” papers to indicate explicitly when functional conservation was experimentally determined. These cross-species expression experiments test whether expressing a putative ortholog from one organism will restore wildtype function to the corresponding inactivated gene in another organism (almost always *S. cerevisiae*). Table 5 summarizes this curated information for only the yeast proteins in the disease-related families to illustrate how this information can be compared to computational results, but P-POD contains experimental results for all yeast proteins for which curated information is available. The orthologs predicted by OrthoMCL often exhibit conserved function. Of the 643 curated complementation experiments between yeast genes and their putative orthologous sequences from other organisms, 395 showed functional conservation and were also identified as orthologs by OrthoMCL; 50 did not complement and were also not predicted to be orthologs by OrthoMCL. Thus, in most cases (445/643), the computational determination of orthology was consistent with experimental results of functional conservation. However, in 153 experiments, complementation was observed, but the proteins were not in the same OrthoMCL family, and in 45 experiments, complementation did not occur, but OrthoMCL predicted an orthologous relationship between the two proteins. These experimental results can be used as a rudimentary assessment of the computational predictions but it must be noted that the definition of orthology does not require functional conservation [10], and there are actual cases (*e.g.* actin) where *in vivo* complementation fails for biological reasons, even for true orthologs that can function *in vitro* [11].

**Table 5:** Functional conservation vs. ortholog prediction: comparing experimental results with the OrthoMCL ortholog predictions for disease-related families.

OrthoMCL	Experimental	Yeast gene	Protein(s) tested	Citation
No	No	*YJL095W: BCK1*	*H. sapiens: ENSP00000306124*	[31]
No	No	*YJR040W: GEF1*	*M. musculus: ENSMUSP00000035964*	[32]
No	No	*YMR190C: SGS1*	*H. sapiens: ENSP00000298139*	[33]
No	No	*YOL090W: MSH2*	*H. sapiens: ENSP00000265081, ENSP00000234420*	[34]
Yes	Yes	*YAL016W: TPD3*	*A. thaliana: AT1G25490.1*	[35]
Yes	Yes	*YBR110W: ALG1*	*H. sapiens: ENSP00000262374*	[36] [37]
Yes	Yes	*YBR140C: IRA1*	*H. sapiens: ENSP00000351015, ENSP00000348498*	[38]
Yes	Yes	*YBR140C: IRA1*	*H. sapiens: ENSP00000351015, ENSP00000352435, ENSP00000348498*	[39]
Yes	Yes	*YBR254C: TRS20*	*H. sapiens: ENSP00000310153*	[40]
Yes	Yes	*YCR075C: ERS1*	*H. sapiens: ENSP00000046640*	[41]
Yes	Yes	*YDL120W: YFH1*	*H. sapiens: ENSP00000297735*	[42], [43]
Yes	Yes	*YDL126C: CDC48*	*A. thaliana: AT3G09840.1*	[44]
Yes	Yes	*YDR270W: CCC2*	*H. sapiens: ENSP00000242839, ENSP00000342559*	[45] [46] [47]
Yes	Yes	*YDR270W: CCC2*	*C. elegans: Y76A2A.2*	[48]
Yes	Yes	*YDR270W: CCC2*	*H. sapiens: ENSP00000343026, ENSP00000345728*	[49] [50]
Yes	Yes	*YDR363W-A: SEM1*	*M. musculus: ENSMUSP00000040741*	[51]
Yes	Yes	*YDR363W-A: SEM1*	*H. sapiens: ENSP00000248566*	[52]
Yes	Yes	*YER095W: RAD51*	*M. musculus: ENSMUSP00000028795*	[53]
Yes	Yes	*YER120W: SCS2*	*H. sapiens: ENSP00000217602, ENSP00000345656*	[54]
Yes	Yes	*YER171W: RAD3*	*H. sapiens: ENSP00000221481*	[55] [56]
Yes	Yes	*YFL018C: LPD1*	*H. sapiens: ENSP00000205402*	[57]
Yes	Yes	*YFR019W: FAB1*	*M. musculus: ENSMUSP00000079926*	[58]
Yes	Yes	*YFR053C: HXK1*	*H. sapiens: ENSP00000338009, ENSP00000223366, ENSP00000350996*	[59]
Yes	Yes	*YGL001C: ERG26*	*M. musculus: ENSMUSP00000033715*	[60]
Yes	Yes	*YGL006W: PMC1*	*A. thaliana: AT2G41560.1*	[61]
Yes	Yes	*YGL006W: PMC1*	*A. thaliana: AT3G21180.1*	[62]
Yes	Yes	*YGL115W: SNF4*	*A. thaliana: AT1G09020.1*	[63], [64]
Yes	Yes	*YGL125W: MET13*	*A. thaliana: AT3G59970.1, AT2G44160.1*	[65]
Yes	Yes	*YGL125W: MET13*	*H. sapiens: ENSP00000315965*	[66]
Yes	Yes	*YGL167C: PMR1*	*H. sapiens: ENSP00000306816, ENSP00000329664, ENSP00000352665*	[67], [68]
Yes	Yes	*YGL167C: PMR1*	*H. sapiens: ENSP00000306816, ENSP00000329664, ENSP00000349901, ENSP00000352580, ENSP00000352665*	[69]
Yes	Yes	*YGL253W: HXK2*	*H. sapiens: ENSP00000338009, ENSP00000223366, ENSP00000350996*	[59]
Yes	Yes	*YGR240C: PFK1*	*H. sapiens: ENSP00000345771, ENSP00000352842*	[70], [71]
Yes	Yes	*YGR267C: FOL2*	*H. sapiens: ENSP00000352686, ENSP00000254299*	[72], [73]
Yes	Yes	*YHR037W: PUT2*	*H. sapiens: ENSP00000290597, ENSP00000336944*	[74], [75]
Yes	Yes	*YIL143C: SSL2*	*A. thaliana: AT5G41360.1*	[76]
Yes	Yes	*YJL059W: YHC3*	*H. sapiens: ENSP00000353116, ENSP00000353116, ENSP00000346650*	[77]
Yes	Yes	*YJL101C: GSH1*	*D. melanogaster: CG2259-PA, CG2259-PB*	[78]
Yes	Yes	*YJR104C: SOD1*	*H. sapiens: ENSP00000270142*	[79]
Yes	Yes	*YJR117W: STE24*	*H. sapiens: ENSP00000196805*	[80], [81]
Yes	Yes	*YJR135W-A: TIM8*	*H. sapiens: ENSP00000247385*	[82], [83]
Yes	Yes	*YKL209C: STE6*	*M. musculus: ENSMUSP00000041204*	[84]
Yes	Yes	*YKL209C: STE6*	*M. musculus: ENSMUSP00000041204, ENSMUSP00000088389*	[85]
Yes	Yes	*YKR079C: TRZ1*	*H. sapiens: ENSP00000337445*	[86]
Yes	Yes	*YLR142W: PUT1*	*A. thaliana: AT5G38710.1*	[87]
Yes	Yes	*YML021C: UNG1*	*H. sapiens: ENSP00000242576, ENSP00000337398*	[88]
Yes	Yes	*YMR190C: SGS1*	*H. sapiens: ENSP00000347232, ENSP00000349859*	[33], [89], [90]
Yes	Yes	*YMR205C: PFK2*	*H. sapiens: ENSP00000345771, ENSP00000352842*	[70], [71]
Yes	Yes	*YNL219C: ALG9*	*H. sapiens: ENSP00000316397*	[36]
Yes	Yes	*YNR030W: ALG12*	*H. sapiens: ENSP00000333813*	[91]
Yes	Yes	*YNR041C: COQ2*	*H. sapiens: ENSP00000310873*	[92]
Yes	Yes	*YNR041C: COQ2*	*A. thaliana: AT4G23660.1*	[93]
Yes	Yes	*YOL049W: GSH2*	*H. sapiens: ENSP00000216951*	[94]
Yes	Yes	*YOL081W: IRA2*	*H. sapiens: ENSP00000351015, ENSP00000348498*	[38], [95]
Yes	Yes	*YOR204W: DED1*	*H. sapiens: ENSP00000310870*	[96]
Yes	Yes	*YOR204W: DED1*	*D. melanogaster: CG9748-PA*	[97]
Yes	Yes	*YPL022W: RAD1*	*A. thaliana: AT5G41150.1*	[98]
Yes	Yes	*YPL153C: RAD53*	*H. sapiens: ENSP00000329178, ENSP00000329012*	[99]
Yes	Yes	*YPL218W: SAR1*	*A. thaliana: AT1G56330.1*	[100]
Yes	Yes	*YPR183W: DPM1*	*S. cerevisiae: DPM1*	[101]
No	Yes	*YBR018C: GAL7*	*H. sapiens: ENSP00000338703*	[102]
No	Yes	*YBR289W: SNF5*	*A. thaliana: AT3G17590*	[103]
No	Yes	*YDR135C: YCF1*	*A. thaliana: AT3G13080.1*	[104], [105]
No	Yes	*YGL006W: PMC1*	*H. sapiens: ENSP00000306816, ENSP00000329664, ENSP00000352665*	[68]
No	Yes	*YGL167C: PMR1*	*A. thaliana: AT1G07810.1*	[106]
No	Yes	*YGL167C: PMR1*	*A. thaliana: AT2G41560.1*	[61]
No	Yes	*YGL167C: PMR1*	*A. thaliana: AT3G21180.1*	[62]
No	Yes	*YHL007C: STE20*	*A. thaliana: AT4G08500.1*	[107]
No	Yes	*YJR040W: GEF1*	*M. musculus: ENSMUSP00000030879*	[32]
No	Yes	*YJR104C: SOD1*	*H. sapiens: ENSP00000307870*	[108]
No	Yes	*YNL098C: RAS2*	*H. sapiens: ENSP00000309845*	[109]
No	Yes	*YOR101W: RAS1*	*H. sapiens: ENSP00000309845*	[109]
No	Yes	*YOR130C: ORT1*	*A. thaliana: AT1G79900.1*	[110]
No	Yes	*YPL111W: CAR1*	*A. thaliana: AT4G08900.1*	[111]
Yes	No	*YDR529C: QCR7*	*H. sapiens: ENSP00000287022*	[112]
Yes	No	*YER148W: SPT15*	*H. sapiens: ENSP00000230354*	[113]
Yes	No	*YNL280C: ERG24*	*D. melanogaster: CG17952-PC*	[114]
Yes	No	*YOL090W: MSH2*	*H. sapiens: ENSP00000233146*	[34]
Yes	No	*YPR183W: DPM1*	*H. sapiens: ENSP00000001585*	[115]

In all but one of these experiments, the yeast gene was mutated and the gene from the other organism was tested for the ability to complement the mutant phenotype. In the one exception, the yeast gene *DPM1* was expressed in mouse. In the OrthoMCL column, “Yes” indicates that the OrthoMCL algorithm placed the two proteins in the same ortholog family, while “No” indicates it did not. In the Experimental column, “Yes” indicates functional complementation, while “No” indicates none. Thus, when both columns are the same, the OrthoMCL prediction is consistent with the experimental result i.e. in the cases where both are “Yes,” the predicted orthologs are functionally conserved, and when both are “No,” the proteins are not predicted to be orthologs, and they are not functionally conserved.

### The P-POD User Interface: Orthologs, Families and Diseases

We designed a simple web interface that allows users to search and browse the data in several ways (Figure 2). Results can be queried by various peptide identifiers or gene names, choosing from any of eight model organisms for the query protein and a particular analysis method, or they can be searched or browsed by Online Mendelian Inheritance in Man (OMIM) ID.

### Searches generate result pages that contain:

- a hyperlinked phylogenetic tree of predicted orthologs generated by OrthoMCL or of more distantly-related proteins generated by Jaccard clustering,
- a list of diseases and genes associated with the human ortholog(s) as documented in OMIM,
- a manually curated list of papers with cross-complementation experiments involving the yeast ortholog(s), and
- a downloadable ClustalW alignment of family members.

### Using P-POD to Compare Methods: Jaccard and OrthoMCL

**Figure 3:**
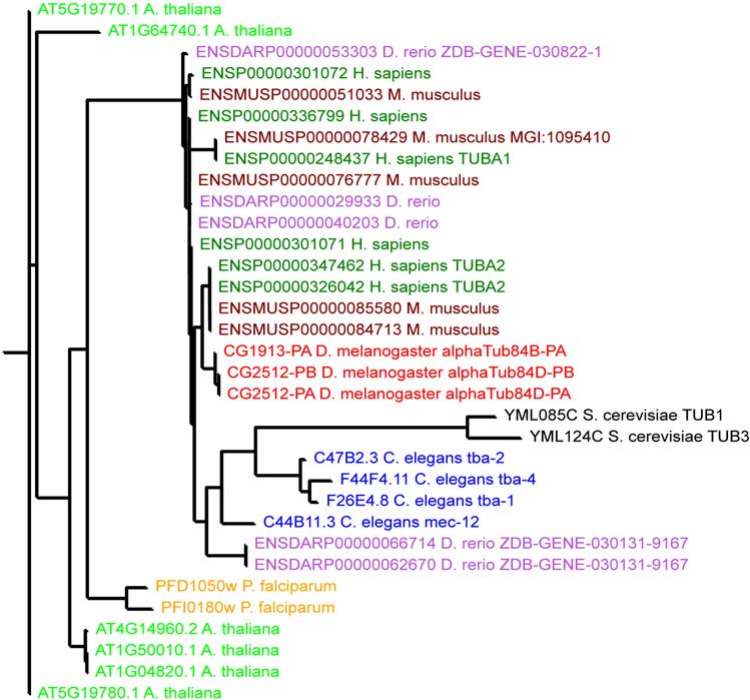
OrthoMCL family of the alpha tubulins. This OrthoMCL family contains only the alpha tubulins, while the tubulin family generated by the Jaccard family (too large to be shown here) contains the alpha, beta, and gamma tubulins.

To illustrate the usefulness of being able to store multiple analyses in a single database, we further compared the results between the OrthoMCL and Jaccard Clustering methods. A query for yeast *TUB1* using only OrthoMCL reveals the alpha tubulins from yeast and other organisms (Figure 3), but not the important paralogous relationships to the beta and gamma tubulins [12] [13], which are observed in the *TUB1* Jaccard cluster (not shown). These three main classes of tubulins are related to the bacterial FtsZ protein and diverged prior to the divergence of the eukaryotes [12]. Many such examples are found, especially among the ancient gene families that go back to the common ancestors of all eukaryotes. The Jaccard clustering provides this larger evolutionary context.

While OrthoMCL identifies predicted orthologs, the Jaccard clustering algorithm should build broader families of more distantly related sequences. Accordingly, one might initially expect that each OrthoMCL family would be a subset of a corresponding Jaccard cluster. Of course, because each algorithm defines homologs quite differently, in practice it would be reasonable to expect a certain degree of disagreement between the OrthoMCL and Jaccard clustering results. Of the 25,271 OrthoMCL families, 17,340 (69%) are subsets of Jaccard clusters. A certain amount of the “loss” of family members is due to stochastic effects; 72% of the 22,216 OrthoMCL families with ten or fewer members remain intact as subsets of Jaccard clusters, compared to only 49% of the 3,055 larger families. Fully 91% of the peptides assigned to OrthoMCL families also lie in Jaccard clusters. 82% of the OrthoMCL families have 80% or more of their peptides in a single Jaccard cluster; 93% have 50% or more.

**Figure 4:**
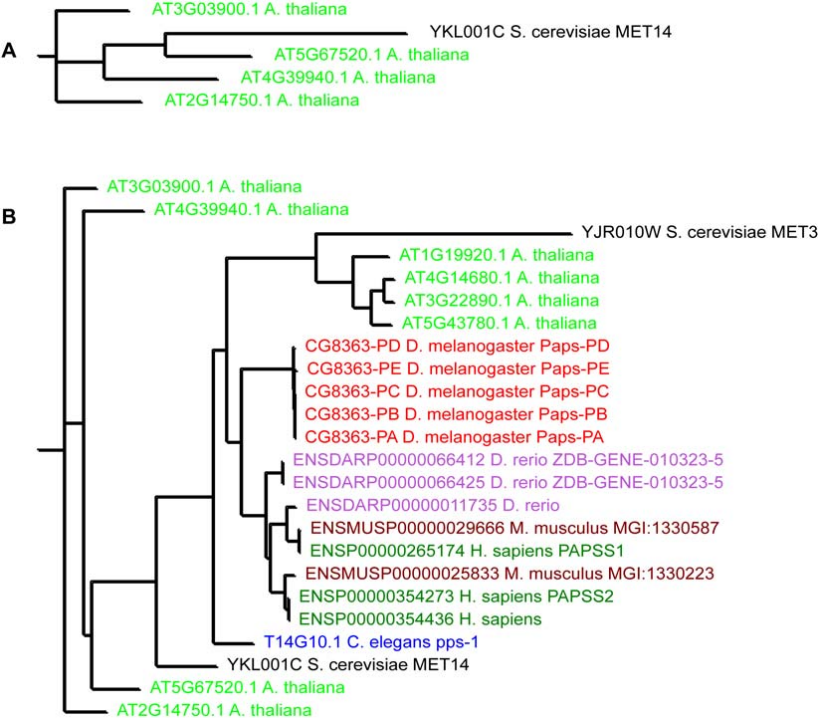
The *MET3/MET14* families. (A) *MET14* Jaccard family, and (B) *MET3/MET14* OrthoMCL family.

Another possible source of inconsistency between the OrthoMCL and Jaccard results is that these analyses were run with different parameter settings. In particular, an alignment constraint was used for the Jaccard clustering alone because the default and recommended settings for OrthoMCL do not include an alignment constraint (see http://orthomcl.cbil.upenn.edu/ORTHOMCL/). The Jaccard clustering software was configured to ignore BLAST hits that did not align over 50% of the length of both peptides. For example, yeast *MET3* and *MET14* respectively encode ATP sulfurylase and adenylylsulfate kinase, which catalyze the first two steps of a sulfate assimilation pathway. *A. thaliana* retains this distinction, but *C. elegans*, *D. melanogaster*, *D. rerio*, human, and mouse have bifunctional proteins containing both activities. The OrthoMCL family contains all of these peptides (Figure 4B), but *MET14* and the four *Arabidopsis* adenylylsulfate kinases form their own Jaccard cluster (Figure 4A). At 202 amino acids, Met14p is less than half the length of the other OrthoMCL family members and therefore fails to satisfy the 50% alignment constraint used in the Jaccard clustering algorithm.

Again, having both sets of results within the same database made comparison of the two methods and detection of possible issues relatively straightforward. We expect that this will be a useful feature for database developers and/or bioinformaticians who may download the entire P-POD system for local installation to use as a development base for their algorithms of choice.

### Other Uses for P-POD

We provide several examples of how P-POD might be used by experimental biologists, and not necessarily those expert in phylogenomics. In addition, we illustrate how providing results from different analysis methods can help to identify issues characteristic of the different methods.

The P-POD system can be used in a simple way to learn something global about the genes and/or proteins of an organism. As an illustration, we studied the conservation of essential genes, *i.e*. genes that are required for viability, across yeast and mammals. Among the 929 OrthoMCL families with unambiguous orthologs from yeast, mouse, and human (*i.e.* exactly one member from each of these species), phenotype data were available for the yeast and mouse genes in 107 cases. In 28 cases, the yeast gene was essential, and in 24 of these families (86%), the mouse gene was also essential. The entire analysis can be found at http://ortholog.princeton.edu/essential_analysis.html.

P-POD can be used to estimate whether essential yeast genes are more likely to be conserved and/or related to a human disease gene. There are 1100 essential and 4670 non-essential yeast genes, respectively. 853 essential yeast genes (77.5%) are found in an OrthoMCL family, while 247 (22.5%) are not. Of the non-essential genes, 2968 (63.6%) are found in families, while 1702 (36.4%) are not. These data suggest that essential genes are more conserved than non-essential genes (χ^2^ = 78, p = 1.1e-18). When examining essentiality among the 954 yeast genes found in disease-related families, 191 of them are essential (20% of the disease-related genes, 17% of all essential genes), while 691 of them are non-essential (72% of disease-related genes, 14.8% of all non-essential genes); phenotype data are not available for the remaining 72 yeast genes. Thus, there does not appear to be enrichment of essential genes among the disease-related yeast genes (χ^2^ = 4.5, p = 0.03). The lack of enrichment of essential genes among disease-related genes is initially surprising; however, this result can be explained if genes required for viability in yeast are also required for viability of human cells, thus making it impossible for the mammal to fully develop into even a diseased organism.

**Figure 5:**
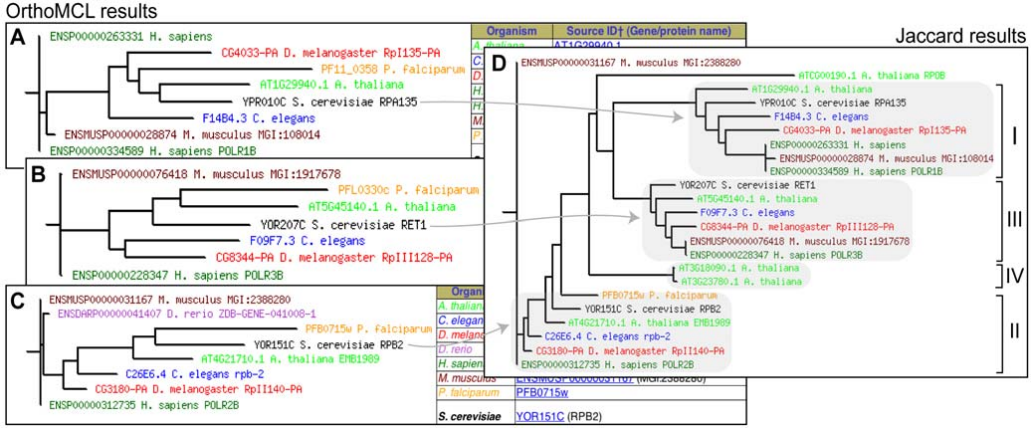
OrthoMCL and Jaccard clustering results for the second largest RNA polymerase subunit families of *S. cerevisiae.* The second largest subunits of RNA polymerase I, II, and III in yeast are named *RPA135, RPB2*, and *RET1*, respectively. (A) Phylogenetic tree display of OrthoMCL results showing individual yeast subunit *RPA135* and its predicted orthologs resolved into a distinct family. OrthoMCL results showing yeast RNA polymerase subunits *RET1* (B) and *RPB2* (C) resolved into separate families of orthologs. (D) Jaccard clustering results showing a “super family” of related RNA polymerase subfamilies. Arrows from each OrthoMCL family on the left point to the separate subfamilies in the Jaccard results. I to IV on the right of each tree indicates RNA polymerase subfamily. The second largest subunits for a fourth RNA polymerase, Pol IV, unique to plants were resolved into their own distinct two-member family by the OrthoMCL program (not shown), and were appropriately clustered with this superfamily by the Jaccard clustering method. (Adapted from figure 2 of [15])

P-POD simplifies the study of the relationships among families of proteins with related functions. One example is the DNA-dependent RNA polymerase family (Figure 5A, B, C). Transcription of genes in eukaryotes is generally performed by three RNA polymerases (I, II, and III), each of which is composed of more than 10 subunits [14], Searching on a selection of individual yeast RNA polymerase subunits (*RPO21, RPO31, RPA190, RPB2, RPB4, RPB5, RPA135*, and *RET1*) resulted in separate phylogenetic tree displays for each protein, demonstrating that they had been effectively resolved into distinct ortholog clusters. Within each cluster, there were mainly one-to-one orthologous relationships between the proteins from each species, except for *RPA135*, and *RET1,* which include orthologs from each species examined except for *D. rerio* (Figure 5A, B).

For some subunits, in particular *RPO21, RPA190*, and *RPA135*, there appear to be more than one mouse or human paralog; however, upon further investigation, it was determined that the separate peptides were encoded by a single mouse or human gene (Figure 5A). Therefore, for the most part, each protein from each species appeared to be orthologous to the others, as would be expected for proteins functioning in a core biological process [14].

Interestingly, experimental evidence shows that although all eukaryotes have RNA polymerases I, II, and III, plants are unique in that they have subunits for a fourth polymerase, Pol IV. The closely related genes, AT3G18090.1 (NRPD2B) and AT3G23780.1 (NRPD2A), have been found to encode the second largest subunit of plant Pol IV, with most of the NRPD2 transcripts coming from NRPD2A. These atypical second largest subunits occurring only in plants are most similar in sequence to the RNA polymerase II second largest subunits in other eukaryotes such as yeast *RPB2* [15], [16]. Despite this sequence similarity, they were effectively resolved away from the OrthoMCL-generated ortholog cluster containing yeast *RBP2* into their own distinct two-member family. The Jaccard clustering method, on the other hand, correctly grouped these unique Pol IV plant subunits with the other second largest RNA polymerase subunit families, as shown in Figure 5D.

As another illustration, we examined thirty yeast ER proteins involved in asparagine-linked glycosylation, a pathway which is well-conserved between yeast and humans in its early steps and diverges soon after glycosylated proteins enter the Golgi (Table 6). Of these, 27 are known from the literature to have human homologs. This analysis shows that 26 lie in ortholog families, with the majority having orthologs in *Homo sapiens* (26), *D. melanogaster* (24), *A. thaliana* (24), *M. musculus* (23), *C. elegans* (23), and *D. rerio* (21). The four proteins that do not lie in ortholog families are subunits of the yeast oligosaccharyltransferase complex. Deleterious mutations in ten of the human homologs cause congenital disorders of glycosylation. Interestingly, only nine of the thirty yeast ER proteins have orthologs in *P. falciparum*. N-linked glycosylation has been detected only at very low levels in *P. falciparum* [17], and ensuring appropriate glycosylation in heterologously-expressed *P. falciparum* proteins has been a technical challenge in the development of malaria vaccines [18], [19].

**Table 6:** Conservation of yeast proteins involved in N-linked glycosylation.

Function	Yeast gene	Human gene	CDG (OMIM)	*At*	*Ce*	*Dm*	*Dr*	*Mm*	*Pf*
**Dolichol synthesis and modification**	*RER2*	DHDDS		x		x	x	x	
	*SEC59*	TMEM15		x	x	x	x		x
	*DPM1*	DPM1	Ie (608799)	x	x	x	x	x	x
	*ALG5*	ALG5		x	x	x	x	x	
	*CAX4*	DOLPP1		x			x	x	x
**Assembly of core oligo-saccharides**	*ALG7*	DPAGT1	Ij (608093)	x	x	x	x	x	x
	*ALG13*	GLT28D1		x	x	x	x	x	x
	*ALG14*	unnamed		x	x	x	x	x	x
	*ALG1*	ALG1	Ik (608540)	x	x	x	x	x	
	*ALG2*	ALG2	Ii (607906)	x	x	x		x	
	*ALG11*	unnamed		x	x	x	x	x	
	*RFT1*	RFT1		x	x	x		x	
	*ALG3*	ALG3	Id (601110)	x	x	x		x	
	*ALG9*	ALG9	Il (608776)	x	x	x	x	x	
	*ALG12*	ALG12	Ig (607143)		x	x	x	x	
	*ALG6*	ALG6	Ic (603147)	x	x	x	x		
	*ALG8*	ALG8	Ih (608104)	x	x	x	x	x	
	*DIE2/ALG10*	ALG10/KCR1		x	x	x			
**Oligo-saccharyl-transferase complex**	*OST1*	RPN1		x	x	x		x	x
	*OST2*	DAD1		x	x	x	x	x	
	*OST3*	TUSC3					x	x	
	*STT3*	ITM1		x	x	x	x	x	x
	*WBP1*	DDOST		x	x	x	x	x	x
**Trimming of outer saccharides**	*CWH41/GLS1*	GCS1	IIb (606056)	x	x	x	x	x	
	*ROT2/GLS2*	GANAB		x	x	x	x	x	
	*MNS1*	MAN1B1		x	x	x	x	x	

Genes are broadly categorized by function. Human genes are identified by name when possible and the corresponding congenital disorders of glycosylation (CDG, with OMIM ID) are shown. For *A. thaliana*, *C. elegans*, *D. melanogaster*, *D. rerio*, *M. musculus*, and *P. falciparum*, boxes marked with “x” indicate that a peptide from this organism was placed in the same OrthoMCL family with the yeast gene. Not shown: *SWP1* is homologous to human ribophorin II [30], and *SWP1, OST4, OST5*, and *OST6* do not lie in ortholog families.

## Discussion

The database system (P-POD) we constructed shows users predicted orthologs of query proteins alone (using OrthoMCL) and in their broader evolutionary context (using Jaccard clustering). It consists of a comparative genomics analysis pipeline whose results are stored in a generic, modular database schema (GMOD/chado) using a freely available database system (PostgreSQL). P-POD is meant not to replace but rather to complement the currently available comparative genomics databases. To our knowledge, no other comparative genomics database provides experimental evidence of conservation curated from the primary literature.

We envision at least three sets of users of our database system. First, molecular biologists can query the database over the web to browse orthology data, both computational and experimental, for their favorite proteins. Another set of users consists of model organism database developers, who will quickly be able to provide comparative genomics tools with their species of interest by implementing our system. Finally, we expect that computational biologists who are developing novel comparative genomics algorithms will find the curated information and computational data from other methods extremely useful in assessing their approach. In addition, by using our system, they will save time in implementation and will be able to more readily distribute their algorithms.

It is important to emphasize that while computational methods to identify orthologs are extremely useful, they are by no means perfect. While OrthoMCL does reasonably well in creating putative orthologous groups, like all computational methods, in many cases it fails, either leaving out true orthologs or inappropriately including paralogs [7]. If one's main goal is to use such an algorithm solely to identify strict orthologs, then the selection of species is critical, and the inclusion of two mammals along with the distantly related *Plasmodium* certainly will increase the number of families that contain extraneous paralogs. Our goal, however, is to provide a database that can serve not only computational or evolutionary biologists but also the day-to-day needs of biologists who work on the common model organisms. P-POD provides a way for biologists to query directly for their gene of interest from their species of study, even though in some cases the phylogenetic trees must be manually examined to determine true orthologs because of the occasional inclusion of paralogs. As more refined methods for automatic detection of orthology are developed (for example, [20], [21]) we plan to incorporate them into the P-POD tool, taking advantage of our modular design scheme.

We plan to provide regular updates to the data contained within the database. At the time of writing, we are running the analysis pipeline with the latest versions of the genomes. In addition, we will add new features to the web interface and will expand upon the amount of data stored within the database. We will also continue to provide curated literature describing experimental confirmation of orthology. All the data within the database are freely and publicly available through the web and by downloading the entire database system via the URL http://ortholog.princeton.edu/.

## Materials and Methods

The overall analysis pipeline is illustrated in Figure 1. The sources and versions of the pipeline components are listed in Table 2.

### WU-BLAST

The same WU-BLAST results were used as input to both OrthoMCL and Jaccard algorithms described below. WU-BLAST (version 2.0MP-WashU) was run with the default BLASTP settings: matrix = BLOSUM62, Expectation Threshold = 10, ctxfactor = 1.0, no filtering.

### OrthoMCL and Jaccard Algorithms

OrthoMCL (v. 1.2, 14-March-2005 [4]) compares the all-against-all BLASTP scores from a set of genomes, first identifying putative orthologs as reciprocal best hits between pairs of genomes, then identifying candidate recent paralogs as proteins within the same species that are more similar to each other than to any sequence in the other species. All orthologs and recent paralogs are then converted into a graph where the nodes represent the proteins and the edges represent their relationships. A normalization step is then used to correct for systematic biases when comparing pairs of genomes. Finally, the ortholog families are resolved by application of the Markov Cluster algorithm (MCL v. 1.005, 05-118). Since this procedure maximally includes in a family only those proteins at least as closely related as between-species reciprocal best hits, the resultant OrthoMCL group can be considered a set of putative orthologs in that every protein in the group is likely orthologous to at least one other group member. Some groups, however, consist solely of proteins from a single species; obviously, such groups only contain recent paralogs, but this information is often of great importance to experimental biologists.

We used the following OrthoMCL parameters. P-value cutoff: 1e^−5^, percent identity and percent match cutoffs: 0, maximum weight: 100.

OrthoMCL family size can be adjusted by changing the inflation index (1.5 in this study), but this does not loosen the fundamental restriction that the algorithm begins with a list of putative orthologs and paralogs. To get larger families showing more distant relationships, we wanted to remove this restriction and include proteins that exhibit significant sequence similarity over a large portion of their lengths. We chose to perform Jaccard clustering and to apply a more broadly-defined set of criteria, namely that members of the same family should have significant BLAST scores over at least half of their length. This last point is important to reduce the chance of grouping two sequences together based on the presence of short promiscuous domains.

In the Jaccard clustering analysis, two proteins are grouped into the same family if they share a significant number of homologs, calculated as follows. First, a list of homologs for each sequence, consisting of those whose relative BLASTP scores are less than 1e^−5^ over a total of at least 50% of the length of each, is generated for each protein. Then the Jaccard index for each pair is calculated; this is the ratio of the magnitude of the intersection of their homolog sets vs. the union, or |A∩B| / |A∪B|. Final clusters are generated by linking proteins whose mutual Jaccard index is above a pre-determined cutoff. We evaluated the impact of varying the cutoff over a range of 0.3 to 0.8 for several well-characterized protein families, such as actins, tubulins, RNA polymerases, and several proteins containing RING finger or SH3 domains. We chose a Jaccard index of 0.4 since it most broadly permitted the inclusion of expected members of the families while excluding obvious non-members. For example, at a cutoff of 0.5, the family containing yeast actin (*ACT1*) inappropriately omitted the human and mouse actin-related proteins *ACTR8* and *Actr8*, while a cutoff of 0.3 was clearly too low and yielded many families with hundreds of extraneous members.

### Generation of phylogenetic trees

P-POD generates phylogenetic trees of the OrthoMCL and Jaccard families using CLUSTAL W [5] and PHYLIP (Felsenstein, J. 2005. PHYLIP (Phylogeny Inference Package) version 3.6. Distributed by the author. Department of Genome Sciences, University of Washington, Seattle), using ProML with global rearrangements turned on. CLUSTAL W was run with the default settings: matrix = BLOSUM, Gapopen = 10, Gapext = 0.2, Gapdist = 8, Max div. = 40, ENDGAPS, NOPGAPS and NOHGAPS off, PWMATRIX = BLOSUM, PWGAPOPEN = 10, PWGAPEXT = 0.1, Distance = Kimura, TOSSGAPS = ON, Output = PHYLIP.

### Literature

During literature curation at SGD for its “Literature Guide” resource, papers may be associated with yeast genes and various topics that describe what the paper addresses. A list of all papers associated with the topics “Cross-species expression” or “Disease-related” was downloaded from the SGD FTP site and loaded into the P-POD database, along with links to the yeast genes as made by the SGD curators. These papers are displayed on the P-POD interface whenever a family that contains the relevant yeast genes is viewed; each paper displayed is hyperlinked to the PubMed database. For the papers associated with the “Cross-species expression” topic, we manually read each paper to extract which gene(s) from which organism(s) were tested, and whether functional complementation was demonstrated. These results are stored in the database and displayed on the P-POD interface.

### Database schema and software

P-POD uses the Generic Model Organism Database (GMOD) database package using PostgreSQL software. Information and documentation about the GMOD schema (also known as the “chado” schema) can be found on the GMOD web site (www.gmod.org). In addition, Supplemental Table 1 (http://ortholog.princeton.edu/help.html#schema) provides details about our particular implementation of the GMOD schema, including how data from our analysis (FASTA files, OrthoMCL results, etc.) are mapped to the GMOD database tables.
